# A Standard Lipid Panel Is Insufficient for the Care of a Patient on a High-Fat, Low-Carbohydrate Ketogenic Diet

**DOI:** 10.3389/fmed.2020.00097

**Published:** 2020-04-15

**Authors:** Nicholas G. Norwitz, Vyvyane Loh

**Affiliations:** ^1^Department of Physiology, Anatomy and Genetics, University of Oxford, Oxford, United Kingdom; ^2^Harvard Medical School, Boston, MA, United States; ^3^Transform Alliance for Health, Newton, MA, United States

**Keywords:** cholesterol, HDL, LDL, Lp(a), ketogenic diet, subfractionation, vitamin C

## Abstract

High-fat, low-carbohydrate ketogenic diets have recently become popular for weight loss and the treatment of numerous chronic diseases; however, the general medical community still expresses concern regarding the impact of high-fat diets on serum lipids and cardiovascular risk. Herein, we report on a young man who adopted a ketogenic diet to treat his inflammatory bowel disease. Incidentally, changes in his serum lipids that would be considered adverse by current standards were noted. A more critical analysis of his lipid profile suggests that the changes he experienced may not be dangerous and may, at least with regard to several parameters, represent improvements. This case study demonstrates how the manner in which lipid panels are often reported and reviewed can lead to misleading conclusions and highlights that, at least in the care of those on a ketogenic diet, more nuanced analyses of lipid subfractionations should be conducted in order for physicians to provide optimal care and clinical recommendations.

## Introduction

Ketogenic diets are high-fat, low-carbohydrate diets that induce the liver to generate ketone bodies, particularly the ketone body β-hydroxybutyrate, a metabolic energy source and signaling molecule evolutionarily designed to efficiently fuel the brain and body during times of carbohydrate scarcity. Although clinical studies implementing ketogenic diets have heretofore been difficult to conduct, and more research is still needed, a body of data demonstrates that ketogenic diets may be useful in the treatment of a wide range of chronic diseases that share inflammation as a common underlying pathology (1–7). One of these diseases is ulcerative colitis, an inflammatory bowel disease. Ketogenic diets may dampen inflammation within the gastrointestinal tract by inhibiting the activity of the NLRP3 inflammasome (8), promoting intestinal stem cell regeneration and gut healing (9), and stimulating the release of bile acids that facilitate intestinal immune system homeostasis (10, 11).

Because ketogenic diets are high-fat, and the still-prevalent lipid heart hypothesis assumes high dietary fat intake causes poor cholesterol profiles and elevated cardiovascular risk, it is medically responsible to follow the serum lipids of individuals who consume ketogenic diets. How rigorously we follow serum lipids matters. Standard lipid panels commonly ordered by practitioners typically only report total cholesterol, HDL-C, LDL-C, and triglycerides. These simple metrics are then used to make clinical recommendations. However, it is now well-established that lipoprotein particles display remarkable heterogeneity in form and function. For example, larger LDL particles appear to be non-atherogenic (12, 13) and different HDL particles secreted by the liver appear to display different antiatherogenic properties in association with their distinct morphologies (14, 15). Furthermore, other lipid parameters that are highly relevant to cardiovascular risk, such as oxidized LDL and Lp(a) measurements, are not typically reported. The standard practice of ordering a simple total cholesterol, HDL-C, LDL-C, and triglycerides panel is likely rationalized by the assumption that there is enough homogeneity in the population for these basic measurements to provide sufficient information to make clinical recommendations. This case study demonstrates that such assumptions may not hold true, particularly in those practicing high-fat, low-carbohydrate ketogenic eating.

## Case Description

The subject is a 24-year-old white Caucasian male with biopsy-confirmed ulcerative colitis, diagnosed at age 21. He exhibits normal blood pressure and a healthy BMI, leads an active lifestyle, and has no history of relevant comorbidities (including diabetes or pre-diabetes), nor confounding lifestyle patterns (including smoking or alcohol abuse). From the time of diagnosis, his ulcerative colitis has been his chief medical complaint. Although his inflammatory bowel disease temporarily went into symptomatic remission with the use of oral mesalamine and prednisone, he began to experience repeated flares several months later. He was subsequently treated with mesalamine and prednisone enemas, both of which also failed to induce remission for more than 3 months. A total of three colonoscopies, as well as measurements of fecal calprotectin, revealed persistent rectosigmoid inflammation. The subject also experienced other inflammatory phenomena during this time, including swollen joints and rosacea, although his hsCRP remained below 3 mg/L. In addition to his prescription medications, the subject adopted and strictly adhered to several diets, including the popular low FODMAP and specific carbohydrate diets, none of which put his disease into lasting remission. At age 23, still with the aim of finding a diet that would ameliorate his gastrointestinal symptoms, the subject adopted a ketogenic diet on his own initiative, with 75–80% of his daily calories derived from fat (~300 g), 15–20% derived from protein (~130 g), and 4–5% derived from carbohydrates (~30 g). His self-reported major fat, protein, and carbohydrate sources included extra virgin olive oil and avocados, seafood, and low-carbohydrate, high-fiber vegetables. Within 1 week of adopting this Mediterranean-style ketogenic diet, his gastrointestinal symptoms improved and his fecal calprotectin dropped from 123 to 19 μg/g, which is within the normal range of <50 μg/g. At the time of this writing 8 months later, the subject has not experienced another colitis flare, his compliance has been monitored by periodic assessment of his blood β-hydroxybutyrate levels, which range between 1.0 and 3.0 mM, and his calprotectin, remeasured on three separate occasions, has remained within the normal range, despite the fact that his has discontinued all of his prescription medications for ulcerative colitis.

**Figure 1:**
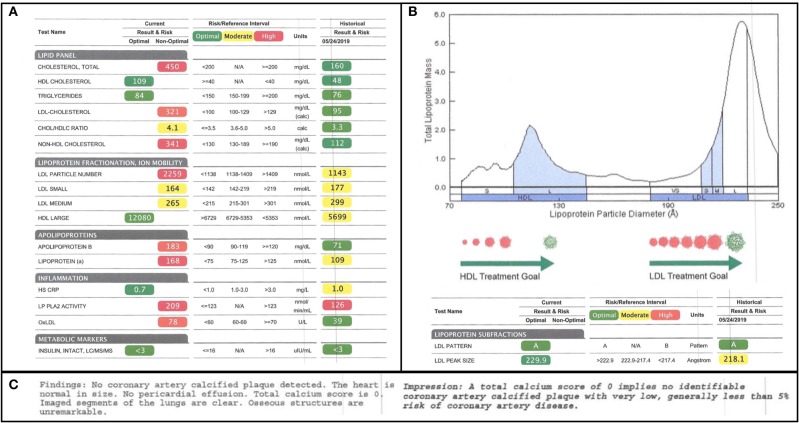
Lipid Subfractionation. **(A)** The subject's baseline lipids, prior to starting his ketogenic diet, are shown on the right. His 7-month follow-up lipids are shown on the left. Green, yellow, and red imply optimal, medium, and high cardiovascular risk, respectively, based on standard reference ranges of isolated variables. **(B)** The subject's lipoprotein size distribution is characterized by a multimodal distribution of HDL, with the greatest peak being in large HDL, and a strong bias in LDL away from atherogenic, small LDL and toward large LDL. **(C)** Results of the subject's coronary artery calcium scan, performed at the time of the 7-month follow-up, indicating little-to-no calcified plaque accumulation.

Given the high-fat nature of the subject's diet, his serum lipids were closely followed. One week prior to starting the diet, a subfractionated lipid panel was drawn and was then repeated 7 months later. Several alarming changes in conventional lipid parameters were flagged in the report in red, as follows: his total cholesterol increased from 160 to 450 mg/dL; his LDL-C increased from 95 to 321 mg/dL; and his LDL-P increased from 1,143 to 2,259 (Figure 1A). These seemingly adverse and dramatic changes were, in small part, offset by an improvement in his HDL-C from 48 to 109 mg/dL. Since total cholesterol, HDL-C, and LDL-C are the cholesterol parameters reported in a standard lipid panel, these are the measures that would typically be used to direct a subject's care. Indeed, according to modifiable risk factor scoring criteria similar to those derived from Framingham but established for individuals ages 15–34, these changes ostensibly represent a four-fold increase in the subject's risk for atherosclerosis (16). However, in this subject, a full fractionated panel was pursued and additionally included a size-based breakdown of the subject's LDL and HDL lipoprotein particles, LDL and HDL particle counts, Apo(B) mass, oxLDL, Lp(a), and PL-PLA2 activity (Figures 1A,B).

Of note, at the time of the 7-month follow-up, a coronary artery calcium scan was also performed as a functional assay of plaque formation. It revealed no significant findings and reported a score of 0, suggesting no signs of calcified atherogenic plaques (Figure 1C).

## Discussion

A standard lipid panel would have revealed that, in this subject, a ketogenic diet induced a large increase in total cholesterol and LDL-C, shifting both parameters from the “optimal” range to more than twice the threshold for the “high-risk” range. Even on the subfractionation, the visuals of the report itself indicated cause for clinical concern: an overwhelming shift in color parameters from normalizing green/yellow to alarming yellow/red (Figure 1). Therefore, it is likely that, were this subject only analyzed by a standard lipid panel, or were the results of the subfractionation not holistically and critically scrutinized, the clinical recommendation would have been that the subject cease his ketogenic diet, a presumptive agent of his colitis remission. Furthermore, this subject may also have been prescribed a statin at 24 years old. Given the mixed evidence on the use of statins for primary prevention and the possibility that long-term statin use in low-risk individuals (including those with a coronary artery calcium score of 0) can contribute to atherosclerosis (17), such a recommendation could have had negative long-term cardiovascular health consequences.

What follows is a more nuanced analysis and discussion of the most relevant changes in the subject's lipid panel, which we suggest do not confer as large an increase in risk as a cursory analysis of certain isolated measures, and the report's color scheme, otherwise imply. An argument could even be made that these changes represent an improvement.

We will start with total cholesterol change and HDL cholesterol as a partial contributor to that change. It is notable that the subject's total cholesterol almost tripled from 160 to 450 mg/dL. Although remarkable, this change alone is not informative without further consideration of what particles are driving the change. A substantial minority of the increase was driven by a doubling of the subject's HDL cholesterol from 45 to 109 mg/dL. Since HDL has antiatherogenic functions, including not only reverse cholesterol transport, but also antioxidant and anti-inflammatory properties, this change is somewhat reassuring. Furthermore, it is generally agreed that, while high HDL-C is associated with good cardiovascular health, HDL-P is a superior predictive measure (14, 15, 18). The subjects large HDL-P jumped from 5,699 nmol/L to a remarkable 12,080 nmol/L. Nearly a dozen separate studies suggest that large HDL particles have a particularly strong association with low cardiovascular risk, even as compared to smaller or medium HDL particles (19–29). However, while there is some disagreement in the field about which HDL particles (small, dense, large, or buoyant) are the most cardioprotective, it has been proposed that it a mix of HDL particles with different morphologies may be ideal (14, 30). This is because, unlike LDL particles, which are secreted in a single form from the liver and decay in size over time, HDL particles are secreted in different forms by the liver and these different forms likely have different functions (30). For example, larger HDL particles may have greater antioxidant capacity, whereas small dense HDL3c may be particularly efficient at reverse cholesterol transport (14, 15). Based on the probable correlation between HDL particles' diverse forms and functions, and the epidemiological data, one could argue that an ideal HDL profile would display a multimodal distribution, one with an overall high particle count with the greatest peak in large HDL-P. This is precisely what is observed in this subject (Figure 1B).

Next, we can examine LDL cholesterol as a major contributor to the subject's total cholesterol increase. Between baseline and follow-up, the subject's LDL-C increased from 90 to 321 mg/dL, the former measure being marked as “optimal,” and the latter, in alarming red, being twice the threshold of “high-risk” (Figure 1A).

However, not all LDL particles are equal. The association between LDL-C and cardiovascular risk is driven by the association between LDL-C and atherogenic small dense and/or oxidized LDL (12, 13). It is primarily the small dense and/or oxidized LDL particles that can penetrate the endothelial wall, be taken up by circulating macrophages, and contribute to foam cell and plaque formation (31, 32). Large LDL particles, by contrast, do not display an association with cardiovascular risk and may, in fact, be cardioprotective (13, 33). A review of the subject's change in LDL-P (from 1,143 to 2,259) and size-based LDL subfractionation reveals that the increase in his LDL is driven exclusively by an increase in large LDL. Both his small and medium LDL even exhibited decreases of 8 and 11%, respectively (Figure 1A).

Since the biological function of LDL is, at least in part, to carry triglycerides from the liver to peripheral tissues as a source of fuel, it is not at all surprising that the subject exhibited an increase in large LDL given his high-fat diet. Furthermore, the fact that only his large LDL increased suggests the subject's large LDL particles did not tend to decay over time into medium and small LDL. Stated more directly, the subject's specific increase in large LDL is consistent with an increase in LDL turnover rate and liver uptake.

This represents a positive and adaptive response to the subject's switching from carbohydrate-based metabolic fuels to fat-based metabolic fuels. This analysis and discussion of LDL metabolism also explains why the increase in LDL-P and Apo(B), both driven by an increase in large LDL-P, may likewise represent healthy and positive adaptions.

Thirdly, we can consider a lesser-known and studied lipoprotein particle, Lp(a). Structurally, Lp(a) is highly similar to LDL except that, appended to Apo-B100, it possesses a glycoprotein tail, apolipoprotein (a). Apolipoprotein (a) itself is remarkably similar in sequence and form to plasminogen, the enzyme that, when activated, binds to and degrades fibrin to break up blood clots. However, apolipoprotein (a) lacks the same protease activity as plasminogen. Lp(a) can thus compete with plasminogen for fibrin binding (inhibiting fibrinolysis) and contribute to the formation of endothelial clots, i.e., atherogenic plaques (34). It therefore makes sense that Lp(a) levels correlate positively with cardiovascular risk (35).

**Figure 2:**
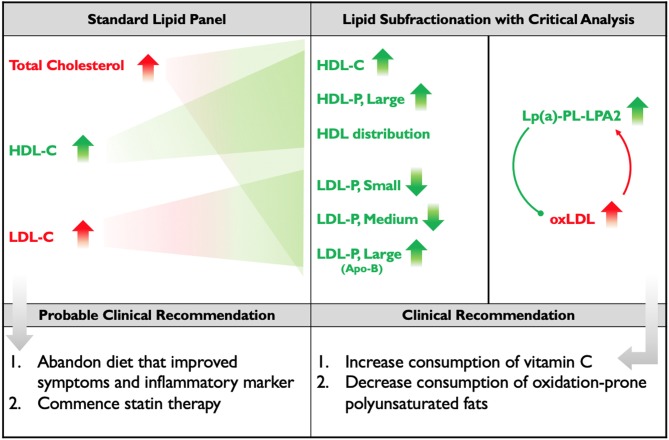
Standard Lipid Panel vs. Lipid Subfractionation. The left column denotes changes induced by the subject's diet that would have been detected on a standard lipid panel, along with the probable course of clinical action. The middle-right column denotes the changes that were detected by subfractionation and, below, the clinical course of action that was recommended. Red indicates changes presumed to be negative; green indicates changes presumed to be positive. Apo(B) is placed in parentheses, below large LDL, because the subject's increase in Apo(B) mass was driven by his increase in large LDL. The diagram within the far-right column illustrates the hypothesis that the subject's elevated Lp(a) and LP-PLA2 activity may represent adaptive, protective responses to the increase in his oxLDL.

Another role for Lp(a) has also been hypothesized, following on the observations that 90% of oxidized lipoproteins (oxLPs) bound to ApoB-containing lipoproteins are actually bound to Lp(a) and that LP-PLA2, an enzyme that degrades atherogenic oxLPs, is associated with Lp(a). It has been proposed that Lp(a)-LP-PLA2 acts as a scavenger for oxLPs (36). Therefore, the subject's increase in Lp(a) and LP-PLA2 activity could both be adaptive responses to the increase in his oxidative status, marked by the increase in his oxLDL (Figure 2).

To interject an added nuance, before returning to the topic of the subject's oxLDL, it possible that his elevated LP-PLA2 activity does not represent a risk at all because (i) on Apo-B-containing particles, LP-PLA2 is most active on small LDL (37, 38) and (ii) HDL-associated LP-PLA2 activity may be antiatherogenic (36, 39, 40). Therefore, this subject's low small-LDL-to-HDL-particle ratio may conceal a cardioprotective factor behind the guise of an atherogenic one.

The increase in the subject's oxLDL is the single change that, in our opinion, is most probably negative. Given that the shift in the subject's diet included an increase in his intake of oxidation-prone polyunsaturated fats (in the form of nuts, seeds, and fatty fish) and a decrease in his intake of antioxidant-containing produce (including vitamin C-containing citrus fruits) it is not entirely surprising that his oxLDL increased. We remark specifically on vitamin C as an antioxidant because another proposed function of Lp(a) is as a surrogate to vitamin C (34). This hypothesis stems from the observations that (i) animals that produce vitamin C endogenously tend not to possess Lp(a) or exhibit heart disease, (ii) there is an inverse correlation between vitamin C status and Lp(a) levels, and (iii) vitamin C is essential in the process of collagen synthesis and endothelial repair. Thus, an evolutionarily adaptive response to insufficient vitamin C would be to increase levels of an antifibrinolytic factor, Lp(a), to induce clot formation and prevent excess bleeding (34).

We therefore hypothesize that were this subject to increase his intake of low-carbohydrate vitamin C-containing foods (such as strawberries, bell peppers, broccoli, and cauliflower) and/or supplement with vitamin C, and also decrease his intake of polyunsaturated fats, exchanging them for more oxidation-resistant monounsaturated fats and possibly some saturated fats that are less likely to impact LDL (including virgin/raw coconut products and stearic acid-rich cacao), he would display a decrease in oxLDL and a consequent decrease in Lp(a) and LP-PLA2 activity (Figure 2). These clinical recommendations have been made, but the subject of this study has relocated and is not currently available for follow-up.

Our report has several limitations. First, no serum cytokines (such as TNF-α and IL-1β) or serum endotoxin were tracked in this patient. It would have been informative to document whether his ketogenic diet improved these markers despite his consistently low hsCRP, as has been reported in other patients adopting ketogenic diets (3). In addition, it is unfortunate that this subject is not currently available for follow-up to document whether the recommended adjustment to his diet altered his Lp(a), LP-PLA2 activity, and oxLDL levels, as we hypothesized. Nevertheless, our analysis of this subject's subfractionation, in conjunction with the functional observation that his coronary artery calcium score is 0, indicating no atherosclerotic plaque formation, argues that the changes in his lipids may not be negative, but rather positive.

## Summary and Significance

Herein, we reported on a subject who adopted a ketogenic diet for ulcerative colitis that successfully put his condition into remission but was also associated with an ostensibly adverse change in his serum lipid profile. A deeper analysis of these lipid profile changes revealed that many parameters might, in fact, be positive. Therefore, rather than recommending the subject abandon the diet that has proved successful in treating his disease, we have recommended a slight nutritional adaptation to see if that optimizes his lipid profile and health.

The significance of this report is threefold: (i) Although clinical anecdotes reveal that ketogenic diets can improve the symptoms of patients struggling with inflammatory bowel diseases, there is little published data on the topic (possibly owing to the high variability among human microbiomes and, thus, patient responsiveness). This report documents an instance in which a ketogenic diet clearly improved a patient's colitis symptoms and laboratory inflammatory markers. (ii) We herein provide data that strongly suggest, at least in the case of subjects on ketogenic diets, standard lipid panels may not be sufficient, and that analyses of lipid subfractionations may be required, in order to inform optimal clinical recommendations. In closing, (iii) this case represents an example of the positive trend in medicine away from formulaic care and toward holistic, personalized, and integrative care.

## Data Availability Statement

All datasets generated for this study are included in the article/supplementary material.

## Ethics Statement

Ethical review and approval was not required for the study on human participants in accordance with the local legislation and institutional requirements. Written informed consent for participation was not required for this study in accordance with the national legislation and the institutional requirements. Written informed consent was obtained from the individual(s) for the publication of any potentially identifiable images or data included in this article.

## Author Contributions

All authors listed have made a substantial, direct, and intellectual contribution to the work, and approved it for publication.

### Conflict of Interest

The authors declare that the research was conducted in the absence of any commercial or financial relationships that could be construed as a potential conflict of interest.

## Abbreviations

Apo(B): apolipoprotein B
HDL-C: HDL content of HDL particles
HDL-P: HDL particle count
hsCRP: high-sensitivity C-reactive protein
LDL-C: LDL content of LDL particles
LDL-P: LDL particle count
Lp(a): lipoprotein(a)
LP-PLA2: lipoprotein-associated phospholipase A2
oxLDL: oxidized LDL.
